# Rapid increase of scrub typhus incidence in Guangzhou, southern China, 2006―2014

**DOI:** 10.1186/s12879-016-2153-3

**Published:** 2017-01-05

**Authors:** Ye Sun, Yue-Hong Wei, Yang Yang, Yu Ma, Sake J. de Vlas, Hong-Wu Yao, Yong Huang, Mai-Juan Ma, Kun Liu, Xiao-Ning Li, Xin-Lou Li, Wen-Hui Zhang, Li-Qun Fang, Zhi-Cong Yang, Wu-Chun Cao

**Affiliations:** 1 The State Key Laboratory of Pathogen and Biosecurity, Beijing Institute of Microbiology and Epidemiology, 20 Dong-Da Street, Fengtai District, Beijing, 100071 People’s Republic of China; 2 Center for Disease Control and Prevention of Jinan Military Region, Shandong Province Jinan, 250014 People’s Republic of China; 3 Guangzhou Center for Disease Control and Prevention, Guangdong Province Guangzhou, 510440 People’s Republic of China; 4 Department of Biostatistics, College of Public Health and Health Professions, and Emerging Pathogens Institute, University of Florida, 32311 Florida, USA; 5 Department of Public Health, Erasmus Medical Center, University Medical Center Rotterdam, 999025 Rotterdam, The Netherlands

## Abstract

**Background:**

In the last decade, scrub typhus (ST) has been emerging or re-emerging in some areas of Asia, including Guangzhou, one of the most affected endemic areas of ST in China.

**Methods:**

Based on the data on all cases reported in Guangzhou from 2006 to 2014, we characterized the epidemiological features, and identified environmental determinants for the spatial distribution of ST using a panel negative binomial model.

**Results:**

A total of 4821 scrub typhus cases were reported in Guangzhou during 2006―2014. The annual incidence increased noticeably and the increase was relatively high and rapid in rural townships and among elderly females. The majority of cases (86.8%) occurred during May―October, and farmers constituted the majority of the cases, accounting for 33.9% in urban and 61.6% in rural areas. The number of housekeeper patients had a rapid increment in both rural and urban areas during the study period. Atmospheric pressure and relative humidity with lags of 1 or 2 months, distributions of broadleaved forest and rural township were identified as determinants for the spatiotemporal distribution of scrub typhus.

**Conclusion:**

Our results indicate that surveillance and public education need to be focused on the elderly farmers in rural areas covered with broadleaf forest in southern China.

**Electronic supplementary material:**

The online version of this article (doi:10.1186/s12879-016-2153-3) contains supplementary material, which is available to authorized users.

## Background

Scrub typhus, a bacterial zoonosis caused by *Orientia tsutsugamushi* (*O. tsutsugamushi*), is characterized by fever, eschar or ulcer, rash, lymphadenopathy, hepatosplenomegaly. Severe complications or even death may occur. *O. tsutsugamushi* is transmitted occasionally to humans by the bites of infected chiggers (larval trombiculid mites) [1, 2]. Rodents are important to maintenance of the disease in that they are known as incidental hosts for chiggers [2]. It is endemic across extensive areas of the Asia-Pacific rim, and over one billion people are at risk to the disease [3]. Recently, a re-emerging picture has been reported from some Asian countries after decades of silence in these regions [4–7]. It was estimated that one million new infections occur worldwide annually, however, which would most likely rise due to the current reemergence in Asia [2]. In the lack of effective human vaccines and convenient and quick diagnostic methods, scrub typhus poses a significant threat to public health [3, 8].

Human cases of scrub typhus were reported in 1948 in Guangzhou. Constantly troubled by the disease, Guangzhou has listed it as one of the local reportable infectious diseases since 1995 [9]. Recently, a rapid increase of the disease was observed with more than one thousand cases reported in 2012, a nearly four-fold increase as compared to 2006 [10]. Scrub typhus in Guangzhou is of the summer-type and is more virulent than the autumn-type scrub typhus which is endemic in Northern China [10]. The increased incidence of scrub typhus in Guangzhou offers an opportunity to enhance our understanding of the epidemiology of this reemerging disease, as well as its spatial and temporal heterogeneity, which will help guide targeted interventions against this disease.

## Methods

### Study area

The study area of Guangzhou prefecture (22°26’ to 23°56’ north latitude, 112°57’ to 114°03’ east longitude), a political subdivision of a province, is the capital of Guangdong Province in southern China. Located at the Pearl River Delta, Guangzhou harbors around 13 million residents in its 12 counties with a total land area of nearly 7500 km^2^ (http://data.gzstats.gov.cn/gzStat1/chaxun/njsj.jsp). It features a subtropical monsoon climate with annual average temperatures of 21–23 °C and annual average precipitations around 1900 mm. The landscape is characterized by mountains and hills in north or northeast, basin and alluvial plain in south or southwest.

### Data collection and management

All clinically diagnosed and laboratory-confirmed cases of scrub typhus are reported to the China Information System for Disease Control and Prevention (CISDCP) since 2006. The diagnosis of scrub typhus is based on the national guide lines issued by the Chinese Center for Disease Control and Prevention (http://www.chinacdc.cn/tzgg/200901/t20090105_40316.htm). The clinical diagnosis of patients were mainly based on some of the following clinical manifestations and signs: (i) Epidemiological exposure history within 3 weeks prior to the onset, (ii) fever, (iii) lymphadenopathy, (iv) skin rash, and (v) specific eschar or ulcer. A clinically diagnosed cases are defined by meeting at least above “i, ii, and v” or “ii, iii, iv, and v” after excluding other diseases with similar clinical manifestations. A laboratory-confirmed case is defined as a clinically diagnosed patient with at least one of the following laboratory results: (vi) An agglutination titer ≥ 1:160 in the Weil-Felix test using the OXK strain of Proteus mirabilis, (vii) a fourfold or greater rise in serum IgG antibody titers between acute and convalescent sera detected by using indirect immunofluorescence antibody assay (IFA), (viii) detection of *O. tsutsugamushi* by polymerase chain reaction (PCR) in clinical specimens, or (ix) isolation of *O. tsutsugamushi* from clinical specimens. A patient meeting above “i, ii, iii or iv” or “ii, iii, iv” after excluding other diseases with similar clinical manifestations, and at least one of above “vii, viii, ix” is also diagnosed as a laboratory-confirmed case according to the Technical Guides for Prevention and Control of Scrub Typhus (available at: http://www.chinacdc.cn/tzgg/200901/t20090105_40316.htm.).

To explore factors influencing the spatiotemporal distribution of scrub typhus cases in Guangzhou, data on meteorological, environmental and ecological factors were collected. The following meteorological data were obtained from China Meteorological Data Sharing Service System (available at: http://data.cma.cn/) : average monthly atmospheric pressure, average monthly temperature, average monthly relative humidity, aggregate monthly precipitation, and average monthly wind velocity. Land cover data were derived from a raster version of the “GlobCover 2009 land cover map” provided by the European Space Agency (available at http://due.esrin.esa.int/page_globcover.php). Elevation raster with a spatial resolution of 1 *1 km^2^ was obtained from the Global Digital Elevation Data Products (http://www.gscloud.cn). Population data at the township level were obtained from the National Bureau of Statistics of China, which is based on the Sixth National Census in 2010. These variables were extracted for each township of Guangzhou in ArcMap version 9.3.

### Epidemiological features analysis

Each reported case was geo-referenced to a digital map of Guangzhou. A thematic map was created to display the case locations and the average annual incidence at township level. Temporal dynamic of the disease for each county were shown by a heat map of monthly incidences from 2006 to 2014. Area feature was classified as rural or urban at township level. For rural and urban areas separately, monthly and annual incidences, and proportions of occupations were plotted over time. Decomposition of annual incidences and average annual incidence by sex and age was also performed, where the average annual incidence was further stratified by urban versus rural areas. In addition, a map series of the incidences at the township level was created for each year from 2006 to 2014.

### Analysis of potential factors associated with the spatial distribution of scrub typhus

A multi-level negative binomial regression model was used to relate the monthly scrub typhus incidences from 2006 to 2014 to potential risk factors at the township level, using the population size as an offset (Additional file 1: Table S1). Variables with *p*-values under 0.10 in the univariate analysis were included in the multivariate analysis. The incidence rate ratio (IRR) in response to the change of each variable by a given amount (Additional file 1: Table S1) was used to show the impact of each variable. Two-sided *p*-values under 0.05 were considered statistically significant. Considering that the mean incubation period of scrub typhus in humans is 10–12 days [11] and that the life cycle of chigger is several months [12], we first examined possible time lags of the influence of each meteorological factor (0–3 months) on monthly incidence of scrub typhus. The most significant lag was then used in the negative binomial regression. Correlations between co-variables were assessed, and highly correlated variables (Spearman correlation coefficients > 0.7) were not entered in the model simultaneously. The multivariate model was selected by comparing the log likelihood of the models and the changes of *p*-values of model coefficients when a covariate was included or excluded [13]. The analysis was performed in STATA 9.1 software (StataCorp LP, CollegeStation TX, USA) [14].

## Results

**Fig. 1:**
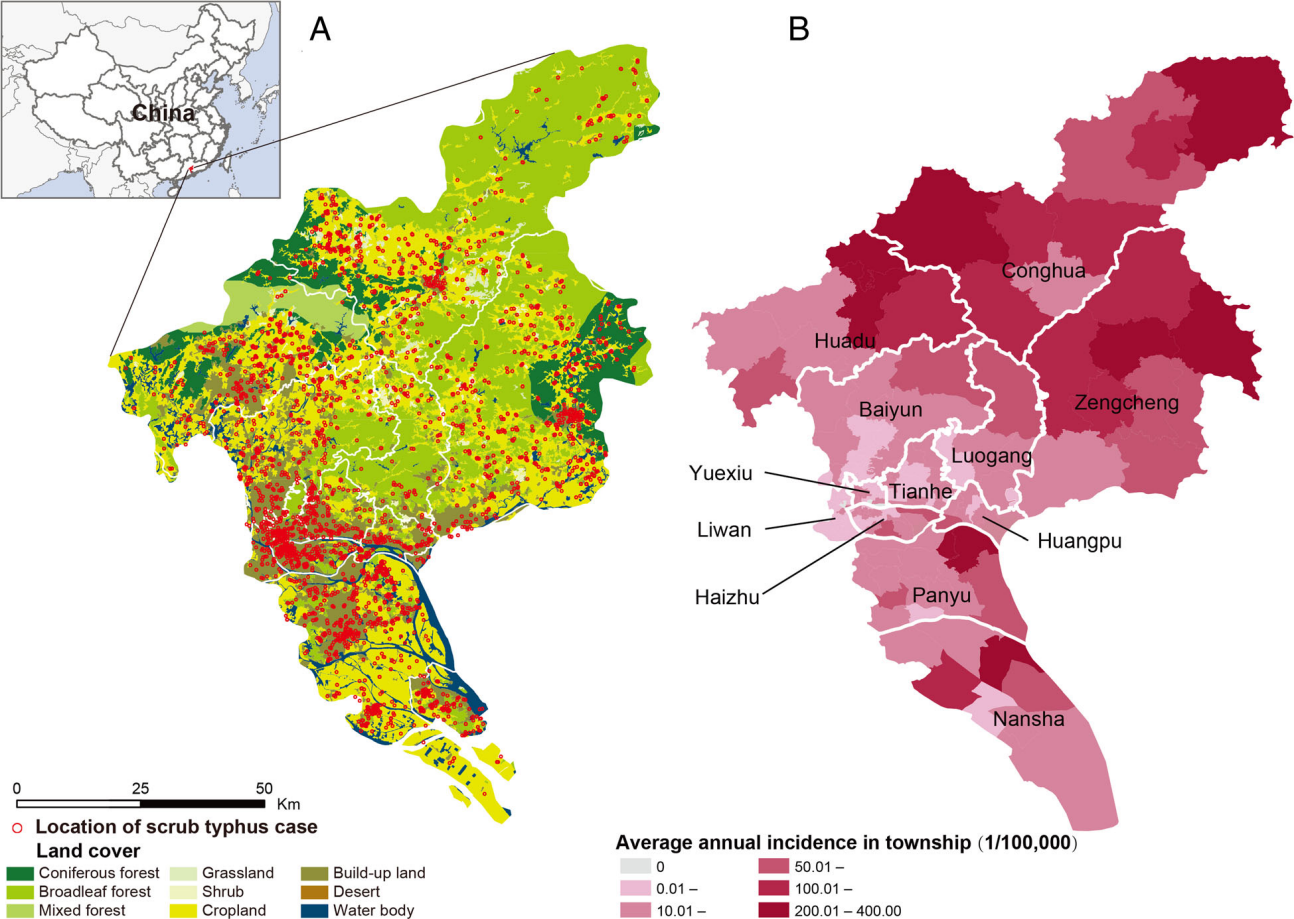
The spatial dynamic of scrub typhus in Guangzhou. **a** Spatial distribution of the locations of confirmed scrub typhus cases overlapped by the land cover, 2006―2014. **b** Average annual incidence of scrub typhus in township, 2006―2014

**Fig. 2:**
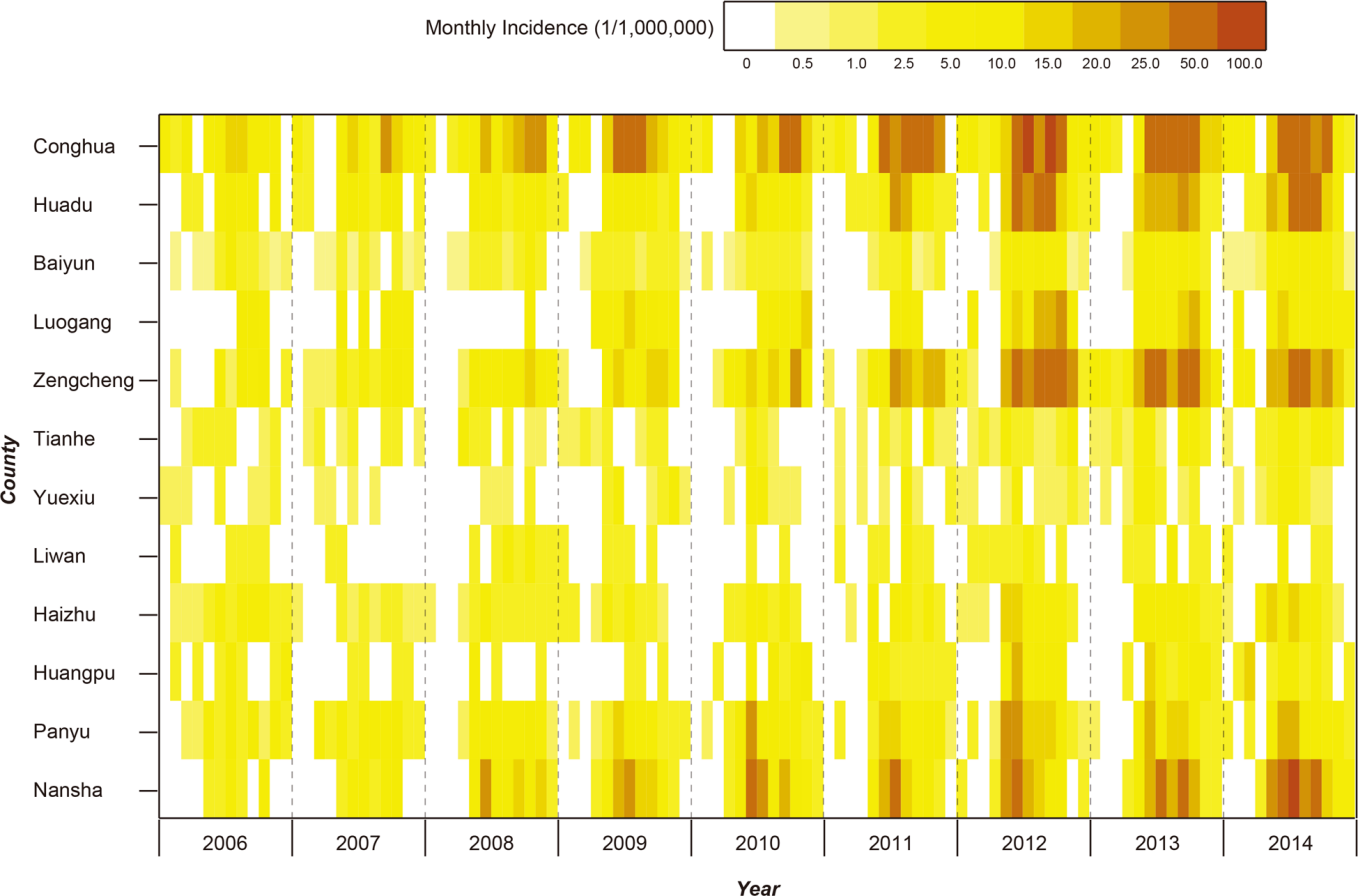
Heat map of monthly incidence of each township in Guangzhou, 2006―2014. Monthly incidences of all townships were shown in the heat map

A total of 4821 clinically diagnosed and laboratory-confirmed cases were reported in Guangzhou during 2006―2014, among whom 14 patients died (CFR: 0.3%). All 12 counties of Guangzhou and 98.8% of the townships (159/161) were affected. Cases were mainly distributed in areas with high densities of built-up lands and croplands (Fig. 1a). The average annual incidences varied greatly across the 159 townships, ranging from 2.2 to 395 per100, 000 person-years, and rural and suburban townships had higher average annual incidences than the urban ones (Fig. 1b). Eleven out of 12 counties showed increasing incidences during the 9 years except for Liwan County in the heat map (Fig. 2). Additional file 2: Figure S1 showed the spatial expansion and distributional dynamics of the disease at the township level.

**Fig. 3:**
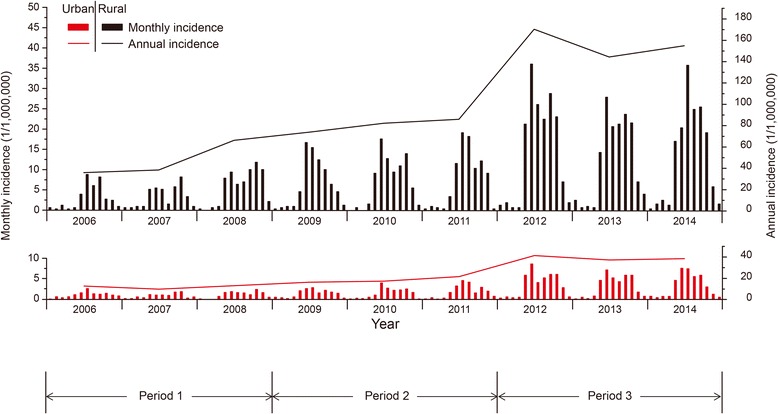
Temporal distribution of monthly scrub typhus incidence for rural and urban areas separately. The bar charts in black and red represent the monthly incidence in rural and urban areas, and the black and red line represents the annual incidence of the scrub typhus in rural and urban areas in Guangzhou, 2006―2014

The majority of cases (86.8%) occurred during May―October, and the incidence peaked in either June/July (most years from 2006―2014) or October (2007―2008) with dual peaks in some years. The annual incidence increased slowly during Period I (2006―2008) and grew steadily during Period II (2009―2011), then fluctuated and remained high in Period III (2012―2014). Rural areas had 2–4 times higher incidences and a more rapid increase of the incidence than urban areas during the study period (Fig. 3). Farmers constitute the majority of the cases over the study period, accounting for 33.9% in urban and 61.6% in rural areas, followed by housekeepers, persons taking housekeeping as their career in their own house or employed by others, who accounted for 19.6% in urban and 12.5% in rural areas. The rapid growth of the number of housekeeper patients was noticeable during the whole period, with an increment of more than 10 times in rural areas and 6 times in urban areas (Additional file 3: Figure S2). Period III was a stage of high growth of the number of cases for housekeepers and farmers, as well as retirees in urban areas.

The senior population older than 60 years had the highest average annual incidence, especially the senior females, which have also shown a quicker rise of average annual incidences in both urban and rural areas during the three periods, rather than other age groups. The age difference in average annual incidence seemed greater in recent period (Additional file 4: Figure S3). Interestingly, in both rural and urban areas, average annual incidences in males were higher than that in females among the population younger than 50, but the direction of gender difference reversed in the older population, i.e., elderly females were more prone to infection with scrub typhus than elderly males.

Univariate negative binomial regressions found that monthly incidences of the disease at the township level were significantly associated with all meteorological variables with 0–2 months lags, various forests, grassland, croplands and artificial surfaces, mean elevation, and type of township (rural *vs.* urban). Four variables, including average atmospheric pressure with 1-month lag, average relative humidity with 2-month lags, percentage coverage of broadleaved forest and type of township, were shown to be independent predictors for the spatiotemporal distribution of the disease in the multivariate regression model. Each one hundred Pa increase of average atmospheric pressure was associated with an 11% (95% CI: 10–12%) decrease in the incidence of scrub typhus in the next month, while a 10% rise in monthly average relative humidity corresponded to an 4% (95% CI: 3–5%) increase in the scrub typhus incidence in the month after the next. For every 10% increase in the percentage coverage of broadleaved forest, the incidence of the disease went up by 5% (95% CI: 3–6%). On average, a rural township had an 81% (95% CI: 43–129%) higher risk than an urban township (Table 1).

**Table 1:** The association between monthly scrub typhus incidence and potential influencing factors by panel negative binomial regression

Variables (unit)^a^	Univariate analysis		Multivariate analysis	
	Crude IRR (95% CI)	*P*-value	Adjusted IRR (95% CI)	*P*-value
AP (1 hPa)	0.87 (0.86, 0.87)	<0.001	0.89 (0.88, 0.90)	<0.001
Temperature (1 °C)	1.17 (1.16, 1.18)	<0.001	NS (excluded)	
RH (10%)	1.11 (1.10, 1.12)	<0.001	1.04 (1.03, 1.05)	<0.001
Precipitation (1 mm)	1.002 (1.002, 1.002)	<0.001	NS (excluded)	
WV (1 m/s)	0.26 (0.22, 0.30)	<0.001	NS (excluded)	
ST (10%)	1.03 (1.03, 1.03)	<0.001	NS (excluded)	
Croplands (10%)	0.97 (0.95, 0.99)	0.001	NS (excluded)	
CV (10%)	0.98 (0.96, 0.99)	<0.001	NS (excluded)	
BF (10%)	1.05 (1.04, 1.06)	<0.001	1.05 (1.03, 1.06)	<0.001
NF (10%)	1.06 (1.04, 1.08)	<0.001	NS (excluded)	
MF (10%)	1.04 (1.02, 1.07)	0.001	NS (excluded)	
GF (10%)	1.15 (1.03, 1.27)	0.012	NS (excluded)	
Shrub (10%)	1.01 (1.01, 1.02)	<0.001	NS (excluded)	
HV (10%)	0.96 (0.95, 0.97)	<0.001	NS (excluded)	
AS (10%)	0.99 (0.99, 0.99)	<0.001	NS (excluded)	
BL (10%)	0.89 (0.84, 0.93)	<0.001	NS (excluded)	
WB (10%)	1.001 (0.997, 1.006)	0.538	-	
Elevation (10 m)	1.09 (1.07, 1.11)	<0.001	NS (excluded)	
FT	1.23 (1.02,1.48)	0.033	1.81 (1.43,2.29)	<0.001

^a^AP, monthly average atmospheric pressure with 1-month lag; Temperature, monthly average temperature with 1-month lag; RH, monthly average relative humidity with 2-month lag; Precipitation, monthly aggregate precipitation with 2-month lag; WV, monthly average wind velocity with 1-month lag; ST, current monthly aggregate proportion of sunlight time; Croplands, percentage coverage of post-flooding or irrigated croplands (or aquatic) and rainfed croplands; CV, percentage coverage of mosaic cropland and vegetation (grassland/shrub/forest); BF, Percentage coverage of broadleaved forest; NF, percentage coverage of needle leaved forest; MF, percentage coverage of mixed broadleaved and needle leaved forest; GF, percentage coverage of mosaic grassland and forest or shrub; Shrub, percentage coverage of broadleaved or needle leaved, and evergreen or deciduous shrub; HV, percentage coverage of herbaceous vegetation (grassland, savannas or lichens/mosses); AS, percentage coverage of artificial surfaces and associated areas; BL, percentage coverage of bared land; WB, percentage coverage of water bodies; Elevation, mean elevation; FT, feature of township (rural vs. urban)

## Discussion

Guangzhou, one of the most affected endemic areas of scrub typhus in mainland China [15], had experienced a rapid increase of scrub typhus incidence during 2006–2014. Our results showed that the hot spots of the disease mainly clustered in rural areas. However, the increasing incidence in urban areas requires more attention because of the much larger population [5, 16]. Some other Asian cities have seen similar increases, for which the increasing outdoor recreation and leisure activities in city parks had been discovered as one of risk factors [17].

The dual peaks, one in June/July and the other in October, were probably linked to the circulation of two different species of mite vectors in the two seasons [10]. The divergent prototypes of *O. tsutsugamushi* identified in Guangzhou could contribute to the dual-peak pattern of this disease [18]. In addition, seasonal changes in the human exposure to mite vectors due to farming or outdoor activities cannot be excluded as a risk factor of the seasonal pattern.

Farmers constituted the majority of the cases. Agricultural activities would increase the exposure to pathogen-carrying chigger mites [19–21]. Notably, about 20–40% of urban cases were also classified as farmers annually, likely a result of the accelerated urbanization in China in recent decades. Residents in newly urbanized areas may still be engaged in a certain level of agricultural activities. Housekeepers were the second largest group of patients and in both rural and urban townships, and the increment of case number was observant (43 and 272 cases during Periods I and III in urban area, and 23 and 257 cases during Periods I and III in rural area, respectively). The majority of the housekeepers were elderly females (>50 years females, 43.2%). The high exposure of housekeepers to infected mites could be associated with their more leisure time and outdoor activities such as walking in parks [22], which should not be overlooked in the planning of intervention programs. The growth of retiree patients in urban areas recently was also noteworthy.

Our data showed that the elder population had higher incidence than the younger during the study period. Also the incidence of rural seniors was rising, especially for females. In modern China, young adults from rural areas often work as laborers in urban areas, leaving the elderly in their hometown taking care of grandchildren as well as farming. More interestingly, elderly females (>50 years) had higher incidence than elderly males, whereas an opposite pattern was observed among the younger generations, regardless of urban or rural townships (Additional file 4: Figure S3). Boys are usually more active in outdoor activities and thus had higher exposure to chigger mites than girls. In rural areas, it is common that elderly males in their fifties or sixties work as laborers in urban cities, but most elderly females stay in their hometown and undertake most of the farming work and housework. Also the keen-on-health Cantonese, especially the elderly, love outdoor exercise, making them more exposed to the disease. The high incidence of the elderly and children could also be attributed to relative low immune level and lack of personal protection.

We found that a higher incidence of scrub typhus was related to the increase of relative humidity at a 2-month lag and the decrease of atmosphere pressure at a 1-month lag. Chigger mites thrive in a moist habitat [23, 24]. Larval population density of chiggers is high in areas of high humidity [23], and chiggers survive and thrive well at relative humidity above 50% [24]. While high atmospheric pressure is thought to be adverse to mites’ survival [9]. The time lags in the effect of meteorological factors may be related to the life cycle of chigger mites as about 2–3 months [12] and the incubation period of the disease (mean of 10–12 days) [11]. Because of the nature of that a chigger normally feeds on its host only once in its life cycle and the infection transmitted by a chigger must have been derived from the female parent by transovarian transmission [12, 25], the infection rate and population density of larval trombiculid mites largely depend on those of the last generation and the habitat during the time between egg hatch and settling down on a host for a larvae, which are influenced by relative humidity and atmosphere pressure in previous about 1–2 months. The contribution of broadleaved forest to the risk of scrub typhus incidence was shown according to our study, where intense substrate vegetative canopies could increase the population density of chigger mites [23]. These factors are similar to those identified in other endemic areas of the disease in Asia [19].

In this study, we characterized the epidemiological features of this reemerging disease in Guangzhou, and identified environmental determinants of the spatial distribution of ST using a panel negative binomial model at township level. However, all the cases used in this analysis were clinically diagnosed and laboratory-confirmed cases based on the national guidelines, and we cannot separate out laboratory-confirmed cases from reported cases. The lack of information on the specific laboratory diagnosis approaches for each reported patient is a limitation of the study. Still, our results provide possible targets for focused prevention and control of ST (i.e., the elderly farmers in rural areas covered with broadleaf forest). Additional experimental studies and the identification of confirmed cases should further explore the epidemic dynamics and influencing factors of ST.

## Conclusions

Our study highlighted the urgent need for prevention programs to contain the spread of scrub typhus in Guangzhou. A set of preventive strategies including public education and personal protection equipment shall be promoted in high-risk populations such as the elderly, farmers and housekeepers. Surveillance and early diagnosis should be reinforced in suburb and rural areas, especially places covered by broadleaf forests. We recommend future studies collect behavioral factors so that risk assessment can be adjusted for individual exposure levels.

## Additional files

Additional file 1: Table S1. Description of potentially influencing factors used in the analysis. (DOCX 19 kb)
Additional file 2: Figure S1. Spatial distribution dynamics of the annual incidence of scrub typhus in Guangzhou, 2006–2014. (DOCX 357 kb)
Additional file 3: Figure S2. The temporal dynamic of the proportion of scrub typhus patients by occupation groups in Guangzhou, 2006–2014. (DOCX 142 kb)
Additional file 4: Figure S3. Age- and gender-specific incidence of scrub typhus in Guangzhou. The star indicates a significant difference of the average incidence between males and females. (DOCX 161 kb)

## Abbreviations

95% CI: 95% confidence interval
CFR: Case fatality rate
CISDCP: China Information System for Disease Control and Prevention
IFA: Indirect immunofluorescence antibody assay
IRR: Incidence rate ratio
*O. tsutsugamushi*: *Orientia tsutsugamushi*
PCR: Polymerase chain reaction
ST: Scrub typhus

## References

[1] Silpapojakul K (1997). Scrub typhus in the Western Pacific region. Ann Acad Med Singapore.

[2] Walker DH (2016). Scrub typhus - scientific neglect, ever-widening impact. N Engl J Med.

[3] Kelly DJ, Fuerst PA, Ching WM, Richards AL (2009). Scrub typhus: the geographic distribution of phenotypic and genotypic variants of Orientia tsutsugamushi. Clin Infect Dis.

[4] Mathai E, Rolain JM, Verghese GM, Abraham OC, Mathai D, Mathai M, Raoult D (2003). Outbreak of scrub typhus in southern India during the cooler months. Ann N Y Acad Sci.

[5] Kweon SS, Choi JS, Lim HS, Kim JR, Kim KY, Ryu SY, Yoo HS, Park O (2009). Rapid increase of scrub typhus, South Korea, 2001–2006. Emerg Infect Dis.

[6] Phongmany S, Rolain JM, Phetsouvanh R, Blacksell SD, Soukkhaseum V, Rasachack B, Phiasakha K, Soukkhaseum S, Frichithavong K, Chu V (2006). Rickettsial infections and fever, Vientiane, Laos. Emerg Infect Dis.

[7] Parola P, Blacksell SD, Phetsouvanh R, Phongmany S, Rolain JM, Day NP, Newton PN, Raoult D (2008). Genotyping of Orientia tsutsugamushi from humans with scrub typhus, Laos. Emerg Infect Dis.

[8] Watt G, Parola P (2003). Scrub typhus and tropical rickettsioses. Curr Opin Infect Dis.

[9] Li T, Yang Z, Dong Z, Wang M (2014). Meteorological factors and risk of scrub typhus in Guangzhou, southern China, 2006–2012. BMC Infect Dis.

[10] Wei Y, Huang Y, Luo L, Xiao X, Liu L, Yang Z (2014). Rapid increase of scrub typhus: an epidemiology and spatial-temporal cluster analysis in Guangzhou City, Southern China, 2006–2012. PLoS One.

[11] Yang LP, Liu J, Wang XJ, Ma W, Jia CX, Jiang BF (2014). Effects of meteorological factors on scrub typhus in a temperate region of China. Epidemiol Infect.

[12] Traub R, Wisseman CL (1968). Ecological considerations in scrub typhus. 2. Vector species. Bull World Health Organ.

[13] Fang LQ, Wang LP, de Vlas SJ, Liang S, Tong SL, Li YL, Li YP, Qian Q, Yang H, Zhou MG (2012). Distribution and risk factors of 2009 pandemic influenza A (H1N1) in mainland China. Am J Epidemiol.

[14] Rabe-Hesketh S, Everitt B (2006). A handbook of statistical analyses using STATA (Third edition).

[15] Zhang WY, Wang LY, Ding F, Hu WB, Soares Magalhaes RJ, Sun HL, Liu YX, Liu QY, Huang LY, Clements AC et al. Scrub typhus in mainland China, 2006–2012: the need for targeted public health interventions. PLoS Negl Trop Dis. 2013, 7(12):e2493. 10.1371/journal.pntd.0002493 PMC3873277 24386495

[16] Park SW, Ha NY, Ryu B, Bang JH, Song H, Kim Y, Kim G, Oh MD, Cho NH, Lee JK (2015). Urbanization of scrub typhus disease in South Korea. PLoS Negl Trop Dis.

[17] Wei Y, Luo L, Jing Q, Li X, Huang Y, Xiao X, Liu L, Wu X, Yang Z (2014). A city park as a potential epidemic site of scrub typhus: a case-control study of an outbreak in Guangzhou, China. Parasit Vectors.

[18] Xia T, Xin-wei W, Yue-hong W, Xin-cai X, Yong Z, Lei L, Jing Z, Fang-hua L (2014). Genotype analysis of orientia tsutsugamushi isolated from humans in Guangzhou in 2012–2013. J Trop Med.

[19] Kuo CC, Huang JL, Ko CY, Lee PF, Wang HC (2011). Spatial analysis of scrub typhus infection and its association with environmental and socioeconomic factors in Taiwan. Acta Trop.

[20] Sharma PK, Ramakrishnan R, Hutin YJ, Barui AK, Manickam P, Kakkar M, Mittal V, Gupte MD (2009). Scrub typhus in Darjeeling, India: opportunities for simple, practical prevention measures. Trans R Soc Trop Med Hyg.

[21] Wardrop NA, Kuo CC, Wang HC, Clements AC, Lee PF, Atkinson PM (2013). Bayesian spatial modelling and the significance of agricultural land use to scrub typhus infection in Taiwan. Geospat Health.

[22] Li T, Yang Z, Wang M. Scrub typhus rapidly increased in Guangzhou, southern China, 2007—2012. Rev Inst Med Trop Sao Paulo. 2013, 55:293–293.

[23] Clopton RE, Gold RE (1993). Distribution and seasonal and diurnal activity patterns of Eutrombicula alfreddugesi (Acari: Trombiculidae) in a forest edge ecosystem. J Med Entomol.

[24] Rubio AV, Simonetti JA (2009). Ectoparasitism by Eutrombicula alfreddugesi larvae (Acari: Trombiculidae) on Liolaemus tenuis lizard in a Chilean fragmented temperate forest. J Parasitol.

[25] Rapsang AG, Bhattacharyya P (2013). Scrub typhus. Indian J Anaesth.

