# Supplementary material for: Rapid increase of scrub typhus incidence in Guangzhou, southern China, 2006―2014
Source: BMC Infect Dis. 2017 Jan 5;17:13. doi: 10.1186/s12879-016-2153-3 (PMC5216553; doi:10.1186/s12879-016-2153-3)
Supplement: Additional file 1: Table S1. — Description of potentially influencing factors used in the analysis. (DOCX 19 kb) [file 12879_2016_2153_MOESM1_ESM.docx]

**Additional Table 1. Description of potentially influencing factors used in the analysis.**

| Variables | Description (Unit) | Type |
| --- | --- | --- |
| AP | Monthly average atmospheric pressure with 1-month lag (1 hPa) | Continuous |
| Temperature | Monthly average temperature with 1-month lag (1 °C) | Continuous |
| RH | Monthly average relative humidity with 2-month lag (10%) | Continuous |
| Precipitation | Monthly aggregate precipitation with 2-month lag (1 mm) | Continuous |
| WV | Monthly average wind velocity with 1-month lag (1m/s) | Continuous |
| ST | Current monthly aggregate proportion of sunlight time (10%) | Continuous |
| Croplands | Percentage coverage of post-flooding or irrigated croplands (or aquatic) and rainfed croplands (10%) | Continuous |
| CV | Percentage coverage of mosaic cropland and vegetation (grassland/shrub/forest) (10%) | Continuous |
| BF | Percentage coverage of broadleaved forest (10%) | Continuous |
| NF | Percentage coverage of needle leaved forest (10%) | Continuous |
| MF | Percentage coverage of mixed broadleaved and needle leaved forest (10%) | Continuous |
| GF | Percentage coverage of mosaic grassland and forest or shrub (10%) | Continuous |
| Shrub | Percentage coverage of broadleaved or needle leaved, and evergreen or deciduous shrub (10%) | Continuous |
| HV | Percentage coverage of herbaceous vegetation (grassland, savannas or lichens/mosses) (10%) | Continuous |
| AS | Percentage coverage of artificial surfaces and associated areas (10%) | Continuous |
| BL | Percentage coverage of bared land (10%) | Continuous |
| WB | Percentage coverage of water bodies (10%) | Continuous |
| Elevation | Average elevation (10m) | Continuous |
| FT | Feature of township (rural *vs.* urban) | Categorical |
