# Supplementary material for: Rapid increase of scrub typhus incidence in Guangzhou, southern China, 2006―2014
Source: BMC Infect Dis. 2017 Jan 5;17:13. doi: 10.1186/s12879-016-2153-3 (PMC5216553; doi:10.1186/s12879-016-2153-3)
Supplement: Additional file 2: Figure S1. — Spatial distribution dynamics of the annual incidence of scrub typhus in Guangzhou, 2006–2014. (DOCX 357 kb) [file 12879_2016_2153_MOESM2_ESM.docx]

**Additional Figure 1. Spatial distribution dynamics of the annual incidence of scrub typhus in Guangzhou, 2006–2014.**

**
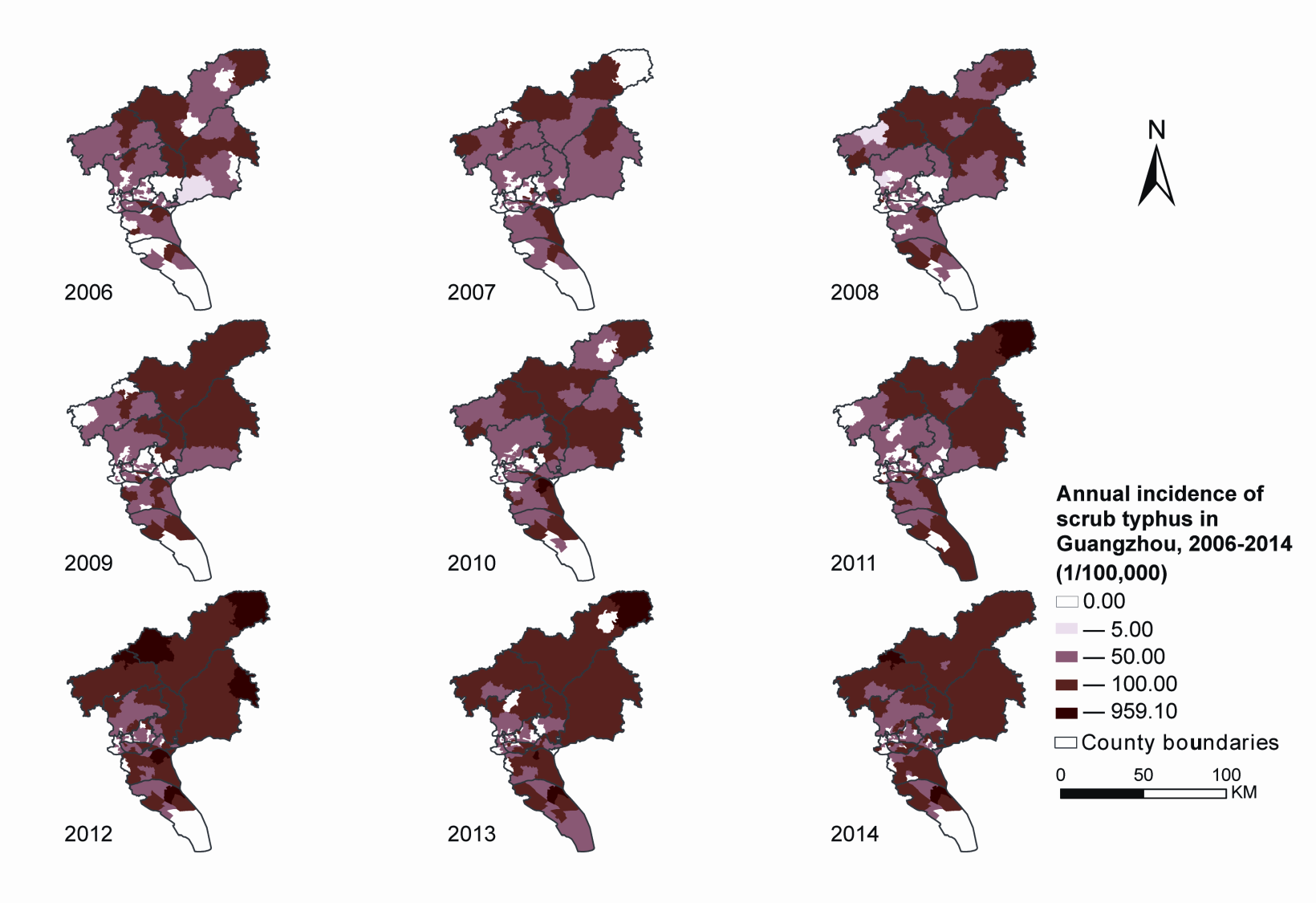
**
