# Supplementary material for: Rapid increase of scrub typhus incidence in Guangzhou, southern China, 2006―2014
Source: BMC Infect Dis. 2017 Jan 5;17:13. doi: 10.1186/s12879-016-2153-3 (PMC5216553; doi:10.1186/s12879-016-2153-3)
Supplement: Additional file 3: Figure S2. — The temporal dynamic of the proportion of scrub typhus patients by occupation groups in Guangzhou, 2006–2014. (DOCX 142 kb) [file 12879_2016_2153_MOESM3_ESM.docx]

**Additional Figure 2. The temporal dynamic of the proportion of scrub typhus patients by occupation groups in Guangzhou, 2006–2014.**

**
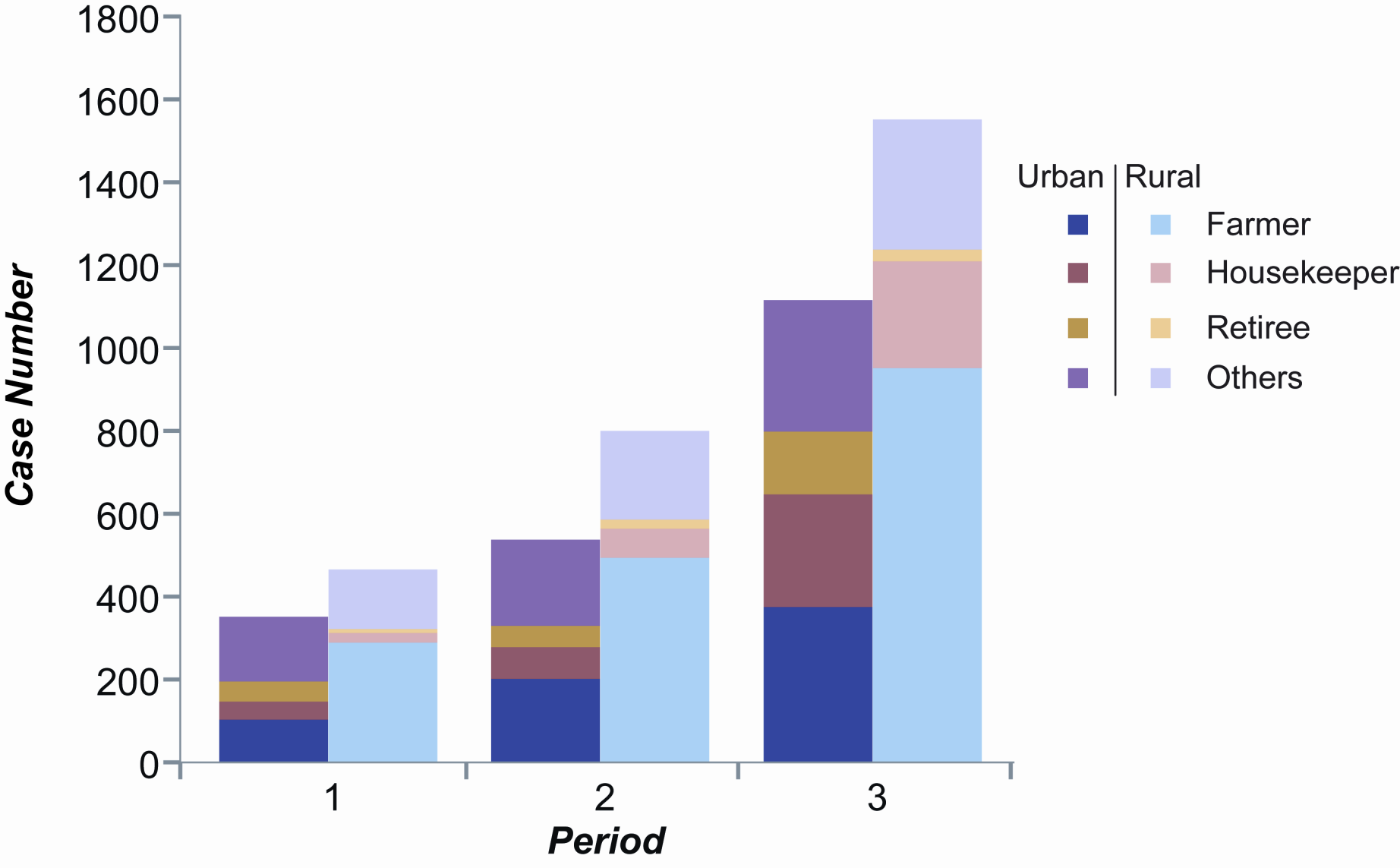
**
