# Supplementary material for: Rapid increase of scrub typhus incidence in Guangzhou, southern China, 2006―2014
Source: BMC Infect Dis. 2017 Jan 5;17:13. doi: 10.1186/s12879-016-2153-3 (PMC5216553; doi:10.1186/s12879-016-2153-3)

**Additional Figure 3. Age- and gender-specific incidence of scrub typhus in Guangzhou.** The star indicates a significant difference of the average incidence between males and females.


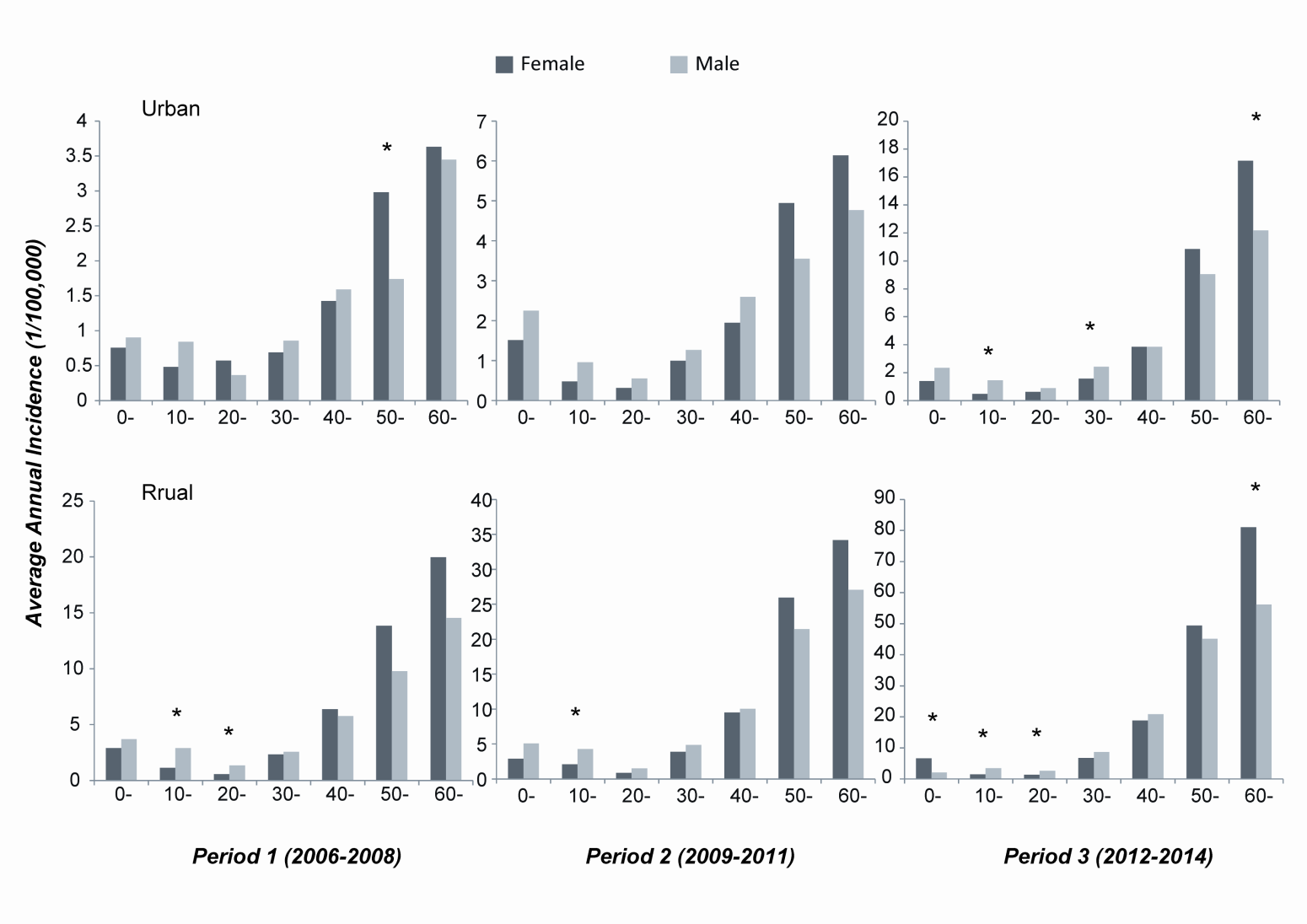

Supplement: Additional file 4: Figure S3. — Age- and gender-specific incidence of scrub typhus in Guangzhou. The star indicates a significant difference of the average incidence between males and females. (DOCX 161 kb) [file 12879_2016_2153_MOESM4_ESM.docx]
